# Dacryoendoscopic recanalization of lacrimal passage obstruction/stenosis after radioiodine therapy for differentiated thyroid carcinoma

**DOI:** 10.1016/j.ajoc.2022.101344

**Published:** 2022-02-03

**Authors:** Daniela Inomata, Sujin Hoshi, Camila Pontes Bessa Campêlo Alcântara, Takahiro Hiraoka, Kuniharu Tasaki, Tetsuro Oshika, Suzana Matayoshi

**Affiliations:** a Department of Ophthalmology, Santa Cruz Hospital, R. Santa Cruz, 398, Vila Mariana, São Paulo, Brazil; b Department of Ophthalmology, Faculty of Medicine, University of Tsukuba, 1-1-1 Tennoudai, Tsukuba City, Ibaraki, Japan; c Department of Ophthalmology, Clinical Hospital, School of Medicine, University of São Paulo (Hospital das Clinicas da Faculdade de Medicina da Universidade de São Paulo – HCFMUSP), Av. Dr. Arnaldo, 455, São Paulo, Brazil

**Keywords:** Nasolacrimal duct obstruction, Radioiodine therapy, Dacryoendoscopy

## Abstract

**Purpose:**

Radioiodine therapy, a standard treatment for differentiated thyroid carcinomas, is associated with several adverse events including lacrimal drainage system obstruction. Herein, we describe the first case of duct lumen recanalization using dacryoendoscopy for lacrimal passage obstruction and stenosis after radioiodine therapy.

**Observations:**

A 48-year-old female treated with radioiodine therapy for differentiated thyroid carcinoma 5 years prior presented with epiphora in both eyes. Dacryocystography showed nasolacrimal duct stenosis in the right eye and nasolacrimal duct obstruction in the left eye. Dacryoendoscopic examination revealed right common canalicular polyps, fibrosis in the right lacrimal sac, right nasolacrimal duct stenosis, and left upper and common canaliculus stenosis. Lacrimal passage recanalization with the insertion of a nasolacrimal stent tube using dacryoendoscopy was performed on the right eye. This successfully resolved the epiphora.

**Conclusions and importance:**

Dacryoendoscopic examination for epiphora after radioiodine therapy may help detect early-stage nasolacrimal passage obstruction/stenosis. This condition can be resolved by recanalization and insertion of a lacrimal tube, without the need for a more invasive surgical approach such as dacryocystorhinostomy.

## 1 Introduction

Radioiodine therapy (RAI) is an established treatment for differentiated thyroid carcinomas^1^ and is commonly used worldwide to treat this increasingly common malignancy.^2^ Ocular complications associated with RAI include chronic and recurrent conjunctivitis, keratoconjunctivitis sicca, and dry eyes, which affect 23% of patients undergoing RAI.^1^ Epiphora and nasolacrimal duct obstruction have also been reported as adverse effects.^3^^,^^4^ Da Fonseca et al.^5^ conducted a prospective study to evaluate the effect of iodine-131 on the lacrimal drainage system. They reported that the rate of lacrimal system obstruction is 6.8% until 12 months after RAI. However, there may be many more lacrimal duct obstruction cases since lacrimal manifestations occur 13–16 months after RAI administration.^3^

Due to the recent and significant evolution of dacryoendoscopy, clearer and more precise images of the duct lumen of the lacrimal passage are achievable.^6^ We describe the first case of duct lumen recanalization using dacryoendoscopy for lacrimal passage obstruction and stenosis after RAI for differentiated thyroid carcinoma.

## 2 Case report

**Fig. 1:**
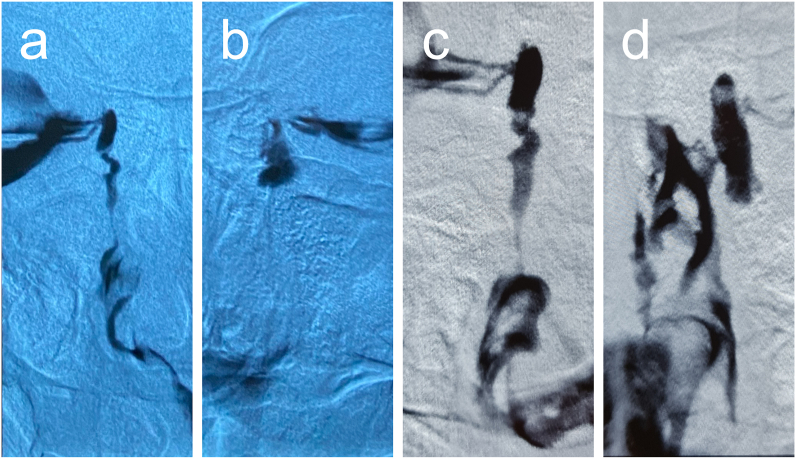
Dacryocystography of the bilateral nasolacrimal duct before and 14 months after surgery. Before surgery, a focal narrowing in the lower part of the nasolacrimal duct on the right side (a) and nasolacrimal obstruction beneath the lacrimal sac on the left side (b) were observed. After surgery, there was still focal narrowing in the lower part of the nasolacrimal duct in the right eye (c), and patency of dacryocystorhinostomy was confirmed in the left eye (d).

A 48-year-old woman presented with epiphora in both eyes. Five years prior, she underwent total thyroidectomy followed by radioiodine therapy at a dose of 200 mCi for differentiated thyroid carcinoma. Three years prior, she noted epiphora with two acute dacryocystitis episodes in the left eye, which were treated with antibiotics at another clinic. She had no history of ocular or lacrimal gland trauma, previous hormone therapy, dysthyroid orbitopathy, and current or previous use of anticancer drugs, such as 5-fluorouracil and docetaxel. The patient's uncorrected visual acuity was 20/20, with intraocular pressure of 12 mmHg on both eyes. Munk scale score, tear meniscus height, lacrimal sac compression test, and lacrimal passage irrigation test suggested epiphora due to lacrimal passage stenosis in the right eye and obstruction in the left eye (Table 1). Dacryocystography showed a focal narrowing in the lower part of the nasolacrimal duct in the right eye (Fig. 1a) and nasolacrimal obstruction beneath the lacrimal sac in the left eye (Fig. 1b).

**Table 1:** Munk scale scores, tear meniscus heights, lacrimal sac compression, and lacrimal passage irrigation test results.

	Right				Left			
	pre	2w	2 m	14 m	pre	2w	2 m	14 m
Munk scale	2	2	0	0	4	1	0	0
Tear meniscus height (mm)	2	1	1	1	4	1	1	1
Lacrimal sac compression test +/−	–	–	–	–	+	–	–	–
Lacrimal passage irrigation test								
Pass +/−	+	+	+	+	–	+	+	+
Reflux +/−	+	+	+	+	+	+	–	–
Pus +/−	–	–	–	–	+	–	–	–

*pre* preoperative, *w* weeks, *m* months.

**Fig. 2:**
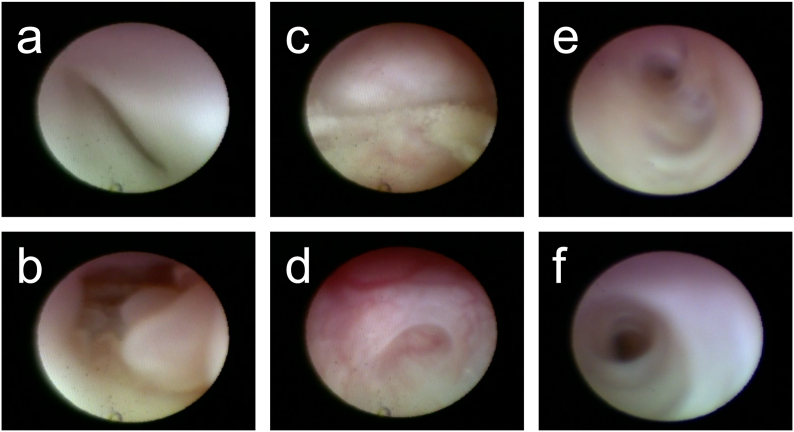
Photograph of dacryoendoscopic examination. a: Right upper canaliculus. Size and mucosa of canaliculus are normal. b: Right common canaliculus. Polyps are observed. c: Right lacrimal sac. The veil-like discharge is observed. d: Lower part of the right lacrimal sac. Fibrosis is observed. e: Severe stenosis is identified in the middle of the nasolacrimal duct. f: Left upper canaliculus. Stenosis is observed.

Dacryoendoscopic examination (LAC-06NZ-HS; MACHIDA Endoscope Co., Ltd., Chiba, Japan) was performed on both eyes under general anesthesia (Fig. 2). The size and mucosal cover of the right upper and lower canaliculus were normal (Fig. 2a). Polyps were observed in the right common canaliculus (Fig. 2b). There were veil-like discharge and fibrosis seen in the right lacrimal sac (Fig. 2c and d). Severe stenosis was seen in the middle of the nasolacrimal duct (Fig. 2e), comparable with the dacryocystographic findings. Nasolacrimal duct recanalization with a lacrimal duct stent tube was performed using dacryoendoscopy using sheath-guided endoscopic probing (SEP) and sheath-guided intubation (SGI) techniques. These techniques enable surgeons to perform lacrimal passage reconstruction under dacryoendoscopic guidance without blind manipulation.^7^

Before SEP, a dacryoendoscope was covered with a sheath that was prepared with an 18-gauge plastic cannula. After the sheath-equipped dacryoendoscope was inserted into the punctum, SEP was performed by widening the stenotic section. After removing the dacryoendoscope, the sheath was temporarily retained in the lacrimal passage and used as a guide for tube insertion during SGI. An 11-cm-long polyurethane Nunchaku-style lacrimal duct stent tube (PF catheter; Toray, Tokyo, Japan) was connected to the sheath. By retrieving the sheath through the nasal cavity, the lacrimal tube was drawn into the recanalized passage. The same procedure was done in the other punctum using a combination of SEP and SGI.

Dacryoendoscopic examination revealed stenosis in the upper canaliculus and severe stenosis in the common canaliculus (Fig. 2f) of the left eye, which interrupted dacryoendoscopy progression into the lacrimal sac. Dacryocystorhinostomy with an external approach was then performed on the left eye.

The Munk scale scores, tear meniscus height, and lacrimal sac compression and irrigation test results during the postoperative period are described in Table 1. The lacrimal tube in the right eye was removed 2 months after intubation. Although reflux was persistently observed during the lacrimal passage irrigation test after lacrimal tube removal in the right eye, the patient experienced epiphora relief and her Munk scale score was zero. The patient's left-eye complaints and clinical findings improved immediately after dacryocystorhinostomy. Recurrence of epiphora or obstruction of lacrimal passage was not observed in both eyes throughout 14 months of observation after surgery. Dacryocystography performed at 14 months after surgery showed that there was still focal narrowing in the lower part of the nasolacrimal duct in the right eye (Fig. 1c), and patency of dacryocystorhinostomy was confirmed in the left eye (Fig. 1d).

This study adhered to the tenants of the Declaration of Helsinki and the patient provided written informed consent for publication.

## 3 Discussion

Lacrimal passage obstruction/stenosis after radioiodine therapy is reported to be located mainly in the nasolacrimal duct, whereas lacrimal passage obstruction/stenosis located in the proximal lacrimal passage is rare.^3^ In the present case, however, lesions in the proximal lacrimal passage, such as common canaliculus polyps, fibrosis in the lacrimal sac, and canaliculus stenosis were observed during the dacryoendoscopic examination. Nasolacrimal duct obstruction is usually more symptomatic and more noticeable when seeking specialized care, which may lead to the underdiagnosis of proximal obstruction/stenosis of the lacrimal system, as is the case here.

Epiphora due to lacrimal passage obstruction can worsen visual function and vision-related quality of life.^8^, ^9^, ^10^ Since patients with epiphora after RAI tend to be around age 50, which seems younger than primary acquired nasolacrimal duct obstruction, the negative effect of epiphora on social activity is a concern.^3^^,^^11^ Dacryoendoscopic examination in patients with epiphora after radioiodine therapy may detect early-stage nasolacrimal obstruction/stenosis, which can be resolved by recanalization and insertion of the lacrimal tube, without the need for a more-invasive surgical approach such as dacryocystorhinostomy.

## 4 Conclusions

The patient in the current case did not undergo postoperative examination with dacryoendoscopy. However, dacryoendoscopic examination can be performed at different times after radioiodine therapy to sequentially document the changes in the lacrimal duct lumen and better understand disease progression.^12^

## Patient consent

The patient consented to publication of the case in writing.

## Funding

This work was supported by the 10.13039/501100006559University of Tsukuba (Intramural Research Promotion Program 2019, Grant No. III-004).

## Authorship

All authors attest that they meet the current ICMJE criteria for Authorship.

## Declaration of competing interest

None.
